# Boosting bulk photovoltaic effect in transition metal dichalcogenide by edge semimetal contact

**DOI:** 10.1038/s41377-024-01691-z

**Published:** 2025-01-02

**Authors:** Shuang Qiao, Jihong Liu, Chengdong Yao, Ni Yang, Fangyuan Zheng, Wanqing Meng, Yi Wan, Philip C. Y. Chow, Dong-Keun Ki, Lijie Zhang, Yumeng Shi, Lain-Jong Li

**Affiliations:** 1 Hebei Key Laboratory of Optic-Electronic Information and Materials, College of Physics Science and Technology, Hebei University, 071002 Baoding, China; 2 Department of Mechanical Engineering, The University of Hong Kong, Hong Kong, China; 3 Department of Physics and HK Institute of Quantum Science & Technology, The University of Hong Kong, Hong Kong, China; 4 Key Laboratory of Carbon Materials of Zhejiang Province, College of Chemistry and Materials Engineering, Wenzhou University, Wenzhou, China; 5 Key Laboratory of Luminescence and Optical Information, Ministry of Education, School of Physical Science and Engineering, Beijing Jiaotong University, 100044 Beijing, China

**Keywords:** Optoelectronic devices and components, Photonic devices

## Abstract

Oxide materials with a non-centrosymmetric structure exhibit bulk photovoltaic effect (BPVE) but with a low cell efficiency. Over the past few years, relatively larger BPVE coefficients have been reported for two-dimensional (2D) layers and stacks with asymmety-induced spontaneous polarization. Here, we report a crucial breakthrough in boosting the BPVE in 3R-MoS_2_ by adopting edge contact (EC) geometry using bismuth semimetal electrode. In clear contrast to the typically used top contact (TC) geometry, the EC metal which strongly adheres to the edges and the subtrates can induce a pronounced tensile strain to the 3R-MoS_2_, and the lateral contact geometry allows to completely access to in-plane polarization from underneath layers reachable by light, leading to >100 times of BPVE enhancement in photocurrent. We further design a 3R-MoS_2_/WSe_2_ heterojunction to demonstrate constructive coupling of BPVE with the conventional photovoltaic effect, indicating their potential in photodetectors and photovoltaic devices.

## Abstract (web summary)

An unprecedented BPVE in 3R-MoS_2_ by utilizing EC Bi semimetal electrodes is observed, and the interplay between the BPVE and PVE is first explored in a specially designed 3R-MoS_2_/WSe_2_ heterojunction.

## Graphical abstract

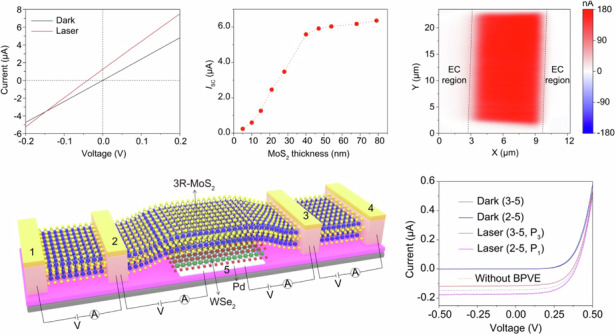

## Introduction

Bulk photovoltaic effect (BPVE), typically observed in materials with a non-centrosymmetric structure, has garnered significant interest due to its potential to surpass the Shockley–Queisser (SQ) limit that governs conventional solar cells^1^. The exploration of the BPVE can be traced back to 1970s, primarily focusing on ferroelectric oxide materials^2,3^. These early studies have made important contributions on exploring the mechanisms underlying the photocurrent generation in the absence of a built-in field^4–6^. However, the photocurrents induced by the BPVE in these materials were generally quite small, and limited to a narrow spectral range owing to their large bandgap energies. Recent studies on two-dimensional (2D) layered materials have suggested that transition metal dichalcogenides (TMDs) hold great promise for harnessing the BPVE^7–15^. The photocurrents observed in TMDs have been found to be orders of magnitude larger than those from other systems^7^. Besides, researchers have pursued various approaches to further enhance the BPVE in TMDs, including strain engineering^7,9,10,13,15^, heterointerfaces^8^, depolarization field^12^, and edge-embedded structure^14^. In particular, 3R-MoS_2_ is with the symmetry that in principle should not exhibit any in-plane polarization and thus no BPVE should be observed. However, once an external strain is applied, the photocurrents observed in 3R-MoS_2_ have been found to present pronounced BPVE^7^. These efforts have yielded significant advancements in understanding and controlling the BPVE in TMDs, thereby paving the way for their applications in efficient photodetectors and photovoltaic devices.

Despite these achievements, the full potential of TMDs in the BPVE has not yet been realized, and several fundamental issues remain to be resolved. One is the typical non-Ohmic contacts between metals and TMDs due to the strong pinning effect of the Fermi levels^16,17^. This makes it difficult to accurately assess the BPVE as the interface Schottky barrier greatly suppresses the currents from BPVE. Noteworthy that, recent findings have shown that semimetal bismuth (Bi) and antimony (Sb) can effectively reduce the metal-TMD Schottky barrier, resulting in ideal Ohmic contacts. This advancement may offer a promising approach to enhance the BPVE in two-dimensional TMDs. The 2^nd^ is that previous devices have typically adopted top contact (TC) electrodes, which may not fully utilize the polarization within TMDs^18,19^. In fact, a very early study on bulk LiNbO_3_ has hinted that the BPVE in the edge contact (EC) configuration is larger than that in the TC configuration^2^. This disparity between TC and EC is expected to be more pronounced in two-dimensional layered TMDs due to their special structures, and is yet to be explored. The 3^rd^ is that the typical way to break the in-plane symmetry for inducing BPVE in these 2D materials is through non-scalable method to add external strain. Moreover, current research on the BPVE primarily focuses on enhancing its photocurrent or efficiency, while the interplay between BPVE and the conventional photovoltaic effect (PVE) remains largely unexplored.

Here, the BPVE in 3R-MoS_2_ was investigated using EC semimetal Bi/Au electrodes. An unprecedented BPVE improvement in both photocurrent and photovoltage was observed owing to the tensile strain induced by the EC Bi metal, low contact resistance of Bi-MoS_2_, and full access of EC to the in-plane polarization from the underlying 3R-MoS_2_ layers. We believe that the strain is primarily induced during the metal deposition stage, where the high deposition temperature facilitates the formation of chemical bonds between the metal and the 3R-MoS_2_ and the tensile strain forms during the cooling owing to the large contraction of metals. Most importantly, the method is simple and without the need to apply additional steps to induce a lateral strain for breaking planar symmetry of 3R-MoS_2_. In addition, by designing a 3R-MoS_2_/WSe_2_ heterojunction, we demonstrate constructive coupling of BPVE with the PVE, contributing to the advancement and potential application in this field.

## Results

**Fig. 1:**
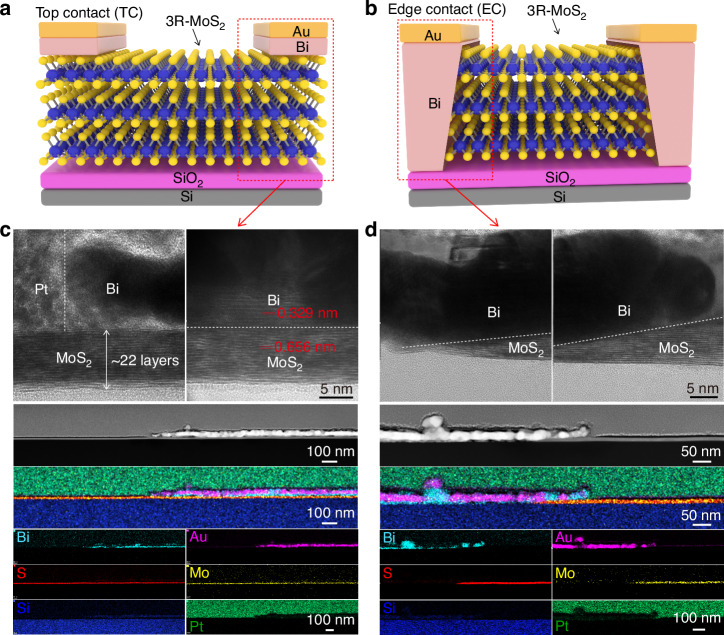
Preparation and characterizations of TC and EC devices. **a**, **b** Schematic illustrations of the TC device (**a**), and the EC device (**b**). **c**, **d** Cross-sectional HRTEM images and STEM-EDS elemental mappings in the right electrode region of the TC device (**c**), and the left electrode region of the EC device (**d**), where Pt is the protection material to support HRTEM imaging

To investigate the difference in BPVE between TC and EC configurations, two types of devices were prepared (Fig. 1a, b), where the processes involved transferring exfoliated 3R-MoS_2_ flakes onto the SiO_2_/Si substrate using polydimethylsiloxane (PDMS). To avoid the flake-to-flake variation, long flakes with uniform widths and thicknesses were chosen, and for fair comparison the TC and EC devices with the same thickness were typically fabricated on the same flake. We used the second harmonic generation (SHG) to determine and make the armchair direction align with the channel direction of the device, as shown in Fig. S1. A standard electron beam lithography (EBL) was used to pattern the electrodes. For the TC device, Bi followed by Au (Bi/Au) was directly deposited on the 3R-MoS_2_ using physical vapor deposition (PVD). While for the EC device, the pre-patterned area was etched with CF_4_ plasma to expose the edges of the 3R-MoS_2_, followed by the Bi/Au deposition. Cross-sectional high-resolution transmission electron microscopy (HRTEM) and energy-dispersive X-ray spectroscopy (EDS) elemental mappings were performed using a scanning transmission electron microscope (STEM) to examine the structures of both TC and EC devices (Fig. 1c, d). The results confirmed the clean interfaces between metals and 3R-MoS_2_. Note that the edges of the 3R-MoS_2_ were not ideally perpendicular to the substrate plane but had a tilt across a length of ~100 nm for a flake of ~22 layers thick, which is limited by the etching technique used in our fabrication process^20^.

**Fig. 2:**
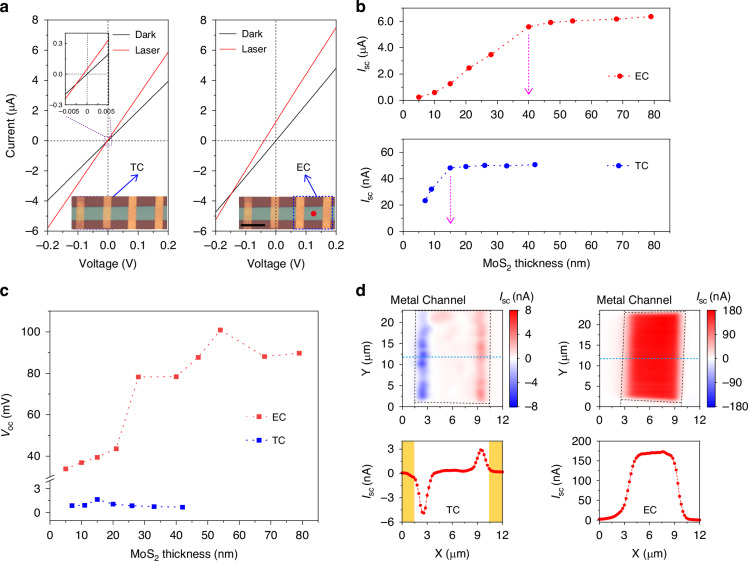
Characteristics of the BPVE in Bi/Au-based 3R-MoS2 devices. **a** The *I–V* curves of the TC and EC 3R-MoS_2_ devices. The laser wavelength is 488 nm and the power is 395 μW. Inset: magnified optical micrographs of the prepared devices with red dots indicating the laser illumination positions for. Scale bars: 10 μm. **b**, **c** 3R-MoS_2_ layer thickness-dependence of the BPVE photocurrent (**b**) and photovoltage (**c**) for both the TC and EC devices. **d** The spatial photocurrent mapping for the TC and EC devices and the illumination position dependences of *I*_sc_ corresponding to the marked dotted light-blue lines. The laser wavelength is 532 nm and the power is 3 μW

To study the effect of contact style on the BPVE of the 3R-MoS_2_, current–voltage (*I–V*) curves were measured under dark and laser illumination conditions (Fig. 2a and S2). A linearly polarized laser (488 nm) with a focused beam diameter of ~1.1 μm was used as the illumination source. During the measurements, the polarization orientation was aligned along the channel direction of the device. Under dark conditions, the TC and EC devices exhibit similar dark current values. However, a significant difference in the BPVE response is observed with light exposure. The TC device exhibits a BPVE short-circuit photocurrent (*I*_sc_) of 48.11 nA and an open-circuit photovoltage (*V*_oc_) of 1.65 mV under a 395 μW power illumination. In sharp contrast, the EC device shows remarkably large *I*_sc_ of 1.26 μA and *V*_oc_ of 39.44 mV. This sharp contrast indicates a substantial enhancement in the BPVE when using the EC configuration compared to the TC configuration. Besides, we also measured the laser polarization-dependent photocurrents for both the EC and TC devices (see Supplementary Fig. S3), where the photocurrents for both exhibit a cosine relation to the laser polarization angle despite the TC shows a much smaller current magnitude^7,8,13^, reinforcing the advantage of the EC in enhancing BPVE. To demonstrate the benefit of using Bi/Au contact, TC and EC devices with typical Cr/Au electrodes were also investigated as a reference (see Supplementary Fig. S4). The dark currents (photocurrents) observed in these devices were more than 100 times (10 times) smaller than those with the Bi/Au electrodes. Despite the reduced BPVE magnitudes, obvious differences between TC and EC devices were still observed. The results evidence that the EC configuration, in combination with the Bi semimetal electrode, can significantly boost the BPVE in the 3R-MoS_2_.

To gain more insights, devices with varied 3R-MoS_2_ thicknesses were studied (see Supplementary Fig. S5, S6 and S7), and the results of *I*_sc_ and *V*_oc_ at an incident power of 395 μW are summarized in Fig. 2b and Fig. 2c. In the EC device, *I*_sc_ exhibits a rapid and linear increase as the thickness of the 3R-MoS_2_ increases from 5 to 40 nm, and then approaches a saturation. The maximum *I*_sc_ value obtained is 6.36 μA, significantly surpassing any previously reported results under illumination with the same laser power level^7–10,13,14^. This thickness-dependent behavior is attributed to the photoabsorption characteristics of the 3R-MoS_2_ (Fig. S8). Based on its absorption coefficient of ~2.57 × 10^5^ cm^−1^ at 488 nm^21^, the calculated penetration depth is ~39 nm, agreeing well with our experimental findings regarding the thickness at which the photocurrent saturates. The result also suggests that the generated carriers can be effectively collected by the EC electrodes. On the other hand, in the TC devices, the *I*_sc_, typically in the range of dozens of nanoamperes, is generally much smaller than that in the EC devices and only shows an increase as the thickness extends to 15 nm, and the *I*_sc_ remains nearly constant with further increase in thickness. This result suggests that the BPVE in TC configuration is limited by the poor interlayer (vertical) carrier transport.

Similar dependence of the *V*_oc_ is also observed since the *V*_oc_ is directly related to the *I*_sc_, as described by the following equation^1,22^:

1 $${V}_{{oc}}=\frac{{J}_{{sc}}l}{{\sigma }_{{ph}}+{\sigma }_{d}}$$

where *l* is the contact distance, *J*_sc_ is the photocurrent density (*I*_sc_ divided by the laser illumination area), and ${\sigma }_{{ph}}$, ${\sigma }_{d}$ is the photoconductivity and the dark conductivity, respectively. Generally, owing to the low *I*_sc_ in TC devices caused by the poor carrier extraction behavior, their *V*_oc_ is very small, in the magnitude of ~1 mV, while the EC devices show significantly higher *V*_oc_, ranging from dozens to hundreds of millivolts.

Spatial distribution measurements of the photocurrent were conducted for both the TC and EC devices to visually distinguish the differences. This was achieved by scanning a focused laser spot over the active device areas (Fig. 2d). In the TC device, a positively low *I*_sc_ of ~0.5 nA was found, distributed almost uniformly throughout the channel region. This observation demonstrates the presence of the BPVE in the 3R-MoS_2_. Additionally, relatively higher photocurrents with an antisymmetric polarity were detected adjacent to the two electrodes, caused by the presence of a small Schottky barrier between metal and 3R-MoS_2_ or photothermal effect. This observation is consistent with previous studies that the Schottky barrier is typically more pronounced than BPVE^8,13^. In clear contrast, the EC device exhibits a homogeneously distributed high *I*_sc_ of ~171.2 nA throughout the device area without the mapping signatures of Schottky barriers at the vicinity of electrodes. This fact again supports the clean edge contact and the depinning effect between the 3R-MoS_2_ and Bi^20^. We have performed the photocurrent mappings for the EC devices with different thicknesses, and the results showed that a similarly homogeneous *I*_sc_ could be obtained throughout the thickness range of 10 to 81 nm (see Supplementary Fig. S9). These spatial distribution measurements provide further evidence of the differences in BPVE between the TC and EC devices, directly highlighting the advantages of the EC configuration. For comparison, we also investigated the BPVE in the EC-based 2H-MoS_2_ devices (see Supplementary Fig. S10). Negligible photocurrents were observed throughout the entire channel regions, indicating that no BPVE was observed in the EC-based 2H-MoS_2_ devices.

In general, the BPVE is suggested to arise from the breaking of inversion symmetry, and such structural asymmetry can be manipulated by factors such as external strain or the creation of heterointerfaces^7,8,13,15^. However, we achieved a significant enhancement in BPVE simply by changing the electrode configuration from TC to EC. It has been previously demonstrated that the metal electrode can naturally induce contact strain on the 3R-MoS_2_ due to the interface chemical bonding and the mismatch in thermal expansion coefficient^23^. This phenomenon is expected to be more pronounced in the EC configuration compared to the TC configuration because the EC metals are directly bonded to the lateral edge of the 2D materials. To verify the presence of contact strain, Raman spectra were conducted, as illustrated in Fig. S11a, b. For the EC device, a uniform redshift of ~0.24 cm^−1^ is observed for the ${E}_{2g}^{1}$ peak in the channel region, as compared to the region outside and far away from the channel (considered as strain-free). This redshift of the points in channel corresponds to a tensile strain at least 0.14%. Note that we estimate the strain using the relation 1.7 cm^−1^ per % strain based on the few-layer MoS_2_ and the thicker layer should in general exhibit a lower Raman shift rate^24^.

Due to the in-plane breaking inversion symmetry of the 3R-MoS_2_ induced by the electrode strain, there should be a strong polarization field, as both theoretically predicted and experimentally observed^7,8,10,13^. Since the lateral transport of carriers within intralayer of TMDs is orders of magnitude faster than the vertical transport of the inter-layer^18,19^, the EC electrodes can efficiently collected the carriers, that is why the effect of EC is much greater than what has been achieved in both the Bi/Au-based TC device (Fig. S12) and the previous Au-based TC device even with a larger external strain^13^. The observation that the photocurrent linearly increases with layer thickness until it reaches a penetration depth of the 3R-MoS_2_ indicates that the contact strain effect and carrier transport process within each layer are quite similar in the EC devices. Hence, the number of collected carriers in the EC device is proportional to the number of the generated carriers. In contrast, no significant Raman redshift can be observed between the point away from metal and those in the Channel (Fig. S11c, d), indicating the absence of substantial contact strain. Moreover, the carriers should experience vertical transport through different layers, which can lead to serious recombination processes along the way. This recombination largely reduces the overall collection efficiency of carriers by the TC electrodes^18,25^, resulting in a very small BPVE response. Regarding the weak thickness dependence of the BPVE, this may be explained by considering the limited interlayer diffusion length in the layered materials. In the case of the TC device, the photo-generated carriers in the bottom layer have a restricted distance over which they can diffuse to reach the top layer metal. The limited inter-layer diffusion length, estimated to be ~15 nm for TMDs^25,26^, limits the influence of the layer thickness on the BPVE response. Moreover, we also performed the contact strain in the EC device of Cr/Au electrodes, as depicted in Fig. S13. The tensile strain of 0.13% observed in the Cr/Au-based device is nearly identical to that in the Bi/Au-based device (0.14%). However, the photocurrent generated through the BPVE effect is approximately 10 times smaller in the Cr/Au-based device. The large difference in photocurrent further confirms the significance of utilizing Bi/Au electrodes to enhance the BPVE efficiency.

Besides, to further verify the presence of a large strain in the EC 3R-MoS_2_, the BPVE photocurrents of EC- and TC-devices have been measured at temperatures ranging from 50 to 325 K, as shown in Fig. S14a, b. For the EC device, the photocurrent exhibits a rapid increase as the temperature decreases. The decrease in temperature leads to a contraction in both the metal electrode and 3R-MoS_2_, causing an increase in the contact strain, which is directly evidenced by the temperature-dependent Raman results, as shown in Fig. S14c, d. While, for the TC device, the photocurrent remains nearly constant across the entire temperature range. This observation further demonstrates that the contact strain in the TC configuration is considerably weaker in comparison, resulting in a negligible temperature-dependent effect on the photocurrent.

We have also explored the effect of adding an external strain to further enhance the BPVE (see Supplementary Fig. S15a). First, we etched a 5 μm-wide groove on a 90 nm-thick h-BN. Then, a layer of 22 nm-thick h-BN was used to pick up and transfer a target 3R-MoS_2_ flake onto the groove to form a clamping structure of top h-BN/3R-MoS_2_/bottom grooved h-BN. By applying additional pressure with the PDMS on the sample, an additional 0.12% uniaxial strain (estimated by Raman spectroscopy^24^ referenced to the original EC device, see Supplementary Fig. S15b) was induced in the groove region. This induces an additional piezoelectric polarization field along the horizontal direction of the 3R-MoS_2_^10,13^, further facilitating the separation of the electron-hole pairs. Detailed measurements can be found in Fig. S15c, d. Despite the 3R-MoS_2_ thickness being only 11 nm and the external applied strain being relatively small, the *I*_sc_ can reach 493 nA (8.68 μA) under 1 μW (395 μW) illumination. These values are considerably larger than the previous results under external strain^9,13^.

**Fig. 3:**
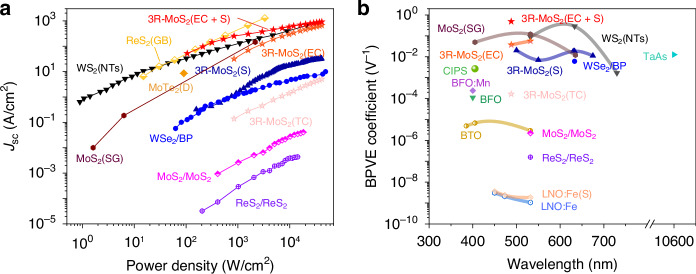
BPVE performance of EC 3R-MoS2 devices and benchmark. **a** The power density-dependence of BPVE *J*_sc_ in reported TMDs. Data for TMDs (WS_2_ nanotubes (WS_2_(NTs))^7^, strain-gradient MoS_2_ (MoS_2_(SG))^9^, strained 3R-MoS_2_ (3R-MoS_2_(S))^13^, WSe_2_/BP heterointerface^8^, Edge-embedded MoS_2_/MoS_2_ and ReS_2_/ReS_2_^14^, distorted MoTe_2_ (MoTe_2_(D))^15^, grain boundary ReS_2_ (GB ReS_2_))^27^ are shown as solid or open symbols and solid lines, respectively. **b** BPVE coefficients for experimented non-centrosymmetric materials. Data for materials (WS_2_(NTs)^7^, MoS_2_(SG)^9^, 3R-MoS_2_(S)^13^, WSe_2_/BP^8^, BiFeO_3_ (BFO)^29^, Mn-doped BFO (BFO:Mn)^29^, TaAs^31^, CuInP_2_S_6_ (CIPS)^32^, BaTiO_3_ (BTO)^33^, Fe-doped LiNbO_3_ (LNO:Fe)^34^, strained LNO:Fe (LNO:Fe(S))^34^) are shown as solid and open symbols. Data for BFO is ${\beta }_{22}$, for BFO:Mn and LNO:Fe are ${\beta }_{33}$, for BTO is ${\beta }_{31}$, and for others are effective values

Figure 3a presents a summary of the short circuit current density (*J*_sc_) versus the laser power density for various TMD materials from existing reports. In our TC device (3R-MoS_2_(TC)), the *J*_sc_ remains low across the entire power density range, although it is still significantly higher than that observed in the edge-embedded MoS_2_/MoS_2_ and ReS_2_/ReS_2_ heterojunctions^14^. Note that the BPVE performance is influenced by both material properties and external conditions. The *J*_sc_ in our EC device (3R-MoS_2_(EC)) using 3R-MoS_2_ is already an order of magnitude larger than that previously achieved in the TC 3R-MoS_2_ even with a larger external strain^13^, and it is even comparable to the best results reported for TMDs^7,27^. Once an additional external strain is further applied to the EC device (3R-MoS_2_(EC + S)), the *J*_sc_ easily exceeds all previous results (especially in the low power density range). Additionally, we have compared our results with other material systems, not limited to TMDs, and the EC 3R-MoS_2_ device consistently demonstrates an absolute advantage in terms of BPVE performance (see Supplementary Fig. S16). Meanwhile, we have also evaluated the BPVE coefficient ($\beta$) based on the relationship between *J*_sc_ and power density (*I*), which is expressed as^28^

$${J}_{{sc}}={\beta }_{{lm}}{E}_{l}{E}_{m}^{* }I$$

where ${E}_{l}$ and ${E}_{m}^{* }$ are the laser polarization unit vectors. As the laser polarization was along the armchair direction during the measurements, the equation can be simplified as^13^

$${J}_{{sc}}={\beta }_{22}I$$

In our TC device, the calculated BPVE coefficient is 1.65 × 10^−4^ V^−1^, which is comparable to the best results achieved in the ferroelectric oxide materials^29^. Moreover, in our EC device, the BPVE coefficient increases to 3.76 × 10^−2 ^V^−1^, and it reaches a record-high value of 4.93 × 10^−1^ V^−1^ after further applying external strain (Fig. 3b).

**Fig. 4:**
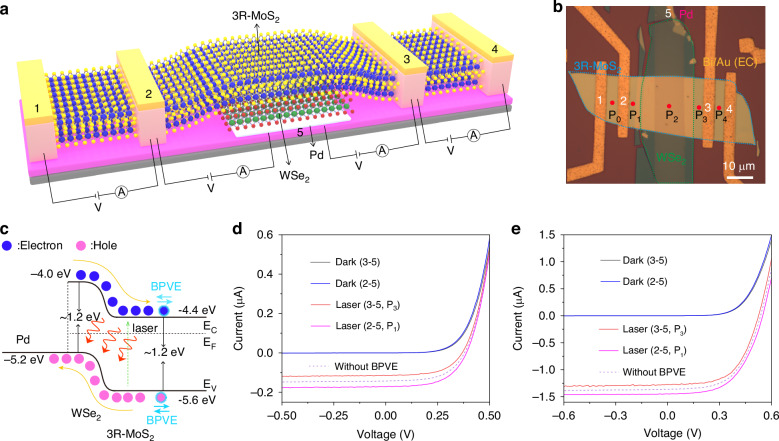
BPVE and PVE performance of EC 3R-MoS2/WSe2 p–n heterojunction. **a** Schematic of the designed EC 3R-MoS_2_/WSe_2_ p–n heterojunction. **b** Optical micrograph of the prepared device. **c**, Band diagram to show the interaction of BPVE and PVE. **d**, **e**, The *I–V* curves of the electrodes 2–5 and 3–5 under dark and laser illumination, when the laser is illuminated on the 3R-MoS_2_ (**d**), and the heterojunction (**e**). Note that based on the results with positive and negative BPVE, the dashed lines are hypothesized as without BPVE. The laser wavelength is 488 nm, and the power is 15 μW

To gain further insights into the effect of the BPVE in conjunction with the traditional photovoltaic effect (PVE), a deliberate design of a 3R-MoS_2_/WSe_2_ p–n heterojunction was fabricated as illustrated in Fig. 4a. First, a 10 nm-thick strip-shaped Pd layer was prepared on a SiO_2_/Si substrate, serving as the bottom electrode. Secondly, a target 3R-MoS_2_ flake was placed orthogonally to a striped-WSe_2_ flake. Then, they were picked up together and transferred to the Pd electrode. Note that the WSe_2_ flake was aligned and fully covered the Pd electrode. Finally, four EC Bi/Au electrodes were prepared on top of 3R-MoS_2_. These electrodes allowed for the measurements of the electrical properties of both the 3R-MoS_2_ and the heterojunction, as depicted in Fig. 4b. This design enables the investigation of the interplay between the BPVE and PVE.

The four electrodes on the 3R-MoS_2_ were numbered as 1, 2, 3, and 4 from left to right, and the bottom Pd electrode was numbered as 5. The laser was illuminated at five different positions on the device (Fig. 4b). Initially, the *I-V* curves were measured between electrodes 1–2 and 3–4 while illuminating the device at positions P_0_ and P_4_, respectively. In both positions, clear BPVE was observed and the *I*_sc_ values were all negative, confirming the left-to-right BPVE current direction (see Supplementary Fig. S17 for pure BPVE effect). On the other hand, if electrode 5 is paired with other electrodes, the photocurrents from the photo-generated electrons and holes shall mainly originate from the built-in field between 3R-MoS_2_ and WSe_2_ heterojunction (PVE). However, due to the presence of BPVE in the 3R-MoS_2_, the polarization field also influences the carrier transport^30^, as illustrated in the band structure of the heterojunction (Fig. 4c). When the photocurrents of BPVE and PVE are in the same direction, an enhancement of the photoresponse should be obtained. Conversely, a reduced photoresponse is expected once they have opposite directions. The dark *I-V* curves exhibited excellent rectification behaviors and almost overlapped for the measurements between electrodes 2–5 and 3–5. Under laser illumination, the electrodes 2–5 and 3–5 exhibited distinct photoresponses. No matter the laser was illuminated on the 3R-MoS_2_ region (P_1_ position for electrodes 2–5 and P_3_ position for electrodes 3–5) or the heterojunction region (P_2_ position for both electrodes 2–5 and 3–5), the PVE responses of electrodes 2–5 were consistently larger than those of electrodes 3–5, which can be explained by the presence of the left-to-right BPVE current. Noteworthy that, under the testing conditions in Fig. 4d and Fig. 4e, the BPVE is estimated to contribute 19.89% and 6.45% to the net PVE photocurrent, respectively (see Supplementary Fig. S18a, b). Moreover, this difference was observed throughout the entire laser power range and increased with higher laser power (see Supplementary Fig. S18c, d). While the differing trends with increasing laser power can be attributed to the distinct PVE responses, along with the contributions from the power-dependent BPVE. This phenomenon was further confirmed by the results obtained from another 3R-MoS_2_/WSe_2_ heterojunction (see Supplementary Fig. S19). The disparity in the photovoltaic response between these two measurements indicates the influence of the BPVE on the overall performance of the heterojunction. This result provides an important insight that the BPVE shows great potential in improving performance of solar cells to break the SQ limit by carefully designing BPVE material-based heterojunctions. Comparative devices based on 2H-MoS_2_/WSe_2_ p–n heterojunction were prepared and studied accordingly. Unlike the 3R-MoS_2_, the 2H-MoS_2_ does not exhibit BPVE due to its centrosymmetric structure^9,13^. As a result, nearly the same PVE responses were obtained for electrodes 1–3 and 2–3 (see Supplementary Fig. S20).

Considering the laser location-sensitive features of the device owing to the presence of BPVE, it can be utilized in the design of a laser position-sensitive detector (PSD) based on the 3R-MoS_2_/WSe_2_ heterojunction. To prove the concept, thirteen equally spaced laser positions were chosen between the 2 and 3 electrodes, with a distance of 2 μm between adjacent positions. By sequentially illuminating the laser on these thirteen positions, the corresponding PVE responses of the electrodes 2–5 and 3–5 were measured. The differing photocurrent values were observed between electrodes 2–5 and 3–5 for reverse symmetric illumination positions (see Supplementary Fig. S21). This information provides valuable insights into the incident laser’s position. Therefore, the PSD based on the 3R-MoS_2_/WSe_2_ heterojunction, combining the BPVE with PVE, holds great potential for applications such as precise laser beam positioning, optical alignment systems and spatial mapping in various fields.

## Discussion

In conclusion, we observed an unprecedented BPVE in 3R-MoS_2_ by utilizing EC electrodes in combination with a near-ideal Ohmic contact of the Bi semimetal. The EC device demonstrated a significantly larger BPVE compared to the TC device, with noticeable improvements in both *I*_sc_ and *V*_oc_, surpassing previously reported values. In addition, the interplay between the BPVE and PVE was explored in a specially designed 3R-MoS_2_/WSe_2_ heterojunction. The contribution of BPVE in the designed heterojunction solar cell device is pronounced. To our knowledge, this is the first observation of coupling those two effects in TMDs, which may lead to various applications.

## Materials and methods

### Device fabrication

The SiO_2_/Si wafer was used as the substrate, with a SiO_2_ thickness of 280 nm. The 3R-MoS_2_, 2H-MoS_2_, WSe_2_, and h-BN crystals were purchased from HQ Graphene. To fabricate the single TC and EC devices, the 3R-MoS_2_ flakes were directly exfoliated onto the SiO_2_/Si substrate using a poly(dimethylsiloxane) (PDMS) sheet. The flakes with suitable thicknesses and shapes were selected using an optical microscope (ZEISS Axioscope 7). Afterward, a uniform poly(methylmethacrylate) (PMMA) layer was spin-coated onto the sample, and the electrodes were patterned using electron-beam lithography (EBL) in the zigzag direction, which was determined by the second harmonic generation (SHG) measurements. Then, a reactive ion etching technique was employed to remove the patterned regions of the flakes with a pure CF_4_ as the working gas, fully exposing the edges of the 3R-MoS_2_. Finally, metal electrodes consisting of a 25–40 nm-thick Bi layer and a 35 nm-thick Au layer) were deposited on the patterned regions using a physical vapor deposition (PVD) technique at a pressure of <2 × 10^−7^ Torr.

In the fabrication process of the strained EC device, the h-BN flakes were exfoliated on the SiO_2_/Si substrate using a PDMS sheet. Both thick and thin flakes with large areas were selected, with the thick h-BN serving as the bottom layer and the thin h-BN as the top layer. To create the desired structure, the thick h-BN was etched similarly to the EC device to obtain a long groove in the center region, with a width of 5 μm. Then, a polycarbonate (PC) covered PDMS dome was employed to pick up the thin h-BN, 3R-MoS_2_ flake sequentially and transfer both of them onto the groove. The flakes were carefully positioned and aligned during this process. To achieve the external strain, the flakes were further pressed down to the bottom of the groove by applying additional pressure using the PDMS. After the flakes were properly positioned, the electrodes were patterned on the flat region of the 3R-MoS_2_, located very close to the groove, etched and evaporated with Bi/Au metal.

To fabricate the 3R-MoS_2_/WSe_2_ heterojunction device, a bottom electrode was first patterned on the SiO_2_/Si substrate and created by depositing Pd metal. Then, the 3R-MoS_2_ and WSe_2_ flakes were exfoliated on the SiO_2_/Si substrates, and suitable flakes were selected using the optical microscope as the target flakes. Using a PC-covered PDMS dome, the selected 3R-MoS_2_ flake was picked up and then stacked on the top of the WSe_2_, ensuring an orthogonal alignment between them. Subsequently, the combined 3R-MoS_2_ and WSe_2_ flakes were picked up together and transferred onto the Pd electrode. Care was taken to align the flakes with the electrode. Finally, EC Bi/Au electrodes were prepared on the top 3R-MoS_2_. The thickness of the 3R-MoS_2_ and WSe_2_ is 17 and 7 nm, respectively.

### Device characterization

The Raman and SHG spectra were measured using a WITec 300 confocal Raman microscopy system with a 1800 g/mm grating. The excitation sources were a 488 nm laser for Raman spectroscopy and a 1064 nm laser for SHG spectroscopy. The thicknesses of the prepared devices were determined using a Bruker Dimension Icon SPM multifunction AFM instrument. TEM was used to examine the device structure at high magnification. A cross-sectional lamella was fabricated using a dual beam focused-ion-beam (FIB) system (Thermo Scientific Helios 5CX). A protective Pt layer was deposited on top of the device to prevent damage during the FIB process. HRTEM images and STEM-EDS elemental mappings were recorded using a Thermo Scientific Talos F200X STEM microscope, operating at 200 kV.

### Photovoltaic performance measurements

Photocurrent measurements were conducted using the confocal Raman microscope, along with a Keysight B1500A source meter. A 488 nm laser was used as the illumination source, and the laser beam was focused on the device using a ×50 objective lens. The laser spot size (ϕ) of ~1.1 μm was calculated based on the equation of ϕ = 1.22 × λ/N.A. (where λ is the laser wavelength, N.A. is the numerical aperture of the objective lens). To ensure consistent measurements, the orientation of the laser polarization was maintained along the device channel. This was achieved by either rotating the device or by using a half-wave plate to adjust the laser polarization orientation. For photocurrent mapping measurements, another commercial measurement system was utilized. A 532 nm laser was used as the illumination source and it was also focused onto the device using a ×50 objective lens. The position of the laser illumination was controlled using a high-precision piezo-stage. Synchronous collection of the photocurrent was achieved using a Keithley 2636B source meter.

## Supplementary information

Supplementary Information

## Data Availability

The data that support the findings of this study are available from the corresponding author upon reasonable request.
