# Supplementary material for: Boosting bulk photovoltaic effect in transition metal dichalcogenide by edge semimetal contact
Source: Light Sci Appl. 2025 Jan 2;14:22. doi: 10.1038/s41377-024-01691-z (PMC11693758; doi:10.1038/s41377-024-01691-z)
Supplement: Supplementary file 1 — Supplementary Information [file 41377_2024_1691_MOESM1_ESM.pdf]

Supplementary Information for Boosting bulk photovoltaic effect in transition metal dichalcogenide by edge semimetal contact

Shuang Qiao[1,2]*[,#], Jihong Liu[1,#], Chengdong Yao[2], Ni Yang[2], Fangyuan Zheng[2], Wanqing Meng[2], Yi Wan[2], Philip C.Y. Chow[2], Dong-Keun Ki[3], Lijie Zhang[4], Yumeng Shi[5], and Lain-Jong Li[2]*

[1]Hebei Key Laboratory of Optic-Electronic Information and Materials, College of Physics Science and Technology, Hebei University, Baoding 071002, China.
[2]Department of Mechanical Engineering, The University of Hong Kong, Hong Kong, China.
[3]Department of Physics and HK Institute of Quantum Science & Technology, The University of Hong Kong, Hong Kong, China.
[4]Key Laboratory of Carbon Materials of Zhejiang Province, College of Chemistry and Materials Engineering, Wenzhou University, Wenzhou, China.
[5]Key Laboratory of Luminescence and Optical Information, Ministry of Education, School of Physical Science and Engineering, Beijing Jiaotong University, Beijing 100044, China.

*Correspondence to sqiao@hbu.edu.cn, lanceli1@hku.hk
[#]These authors contributed equally

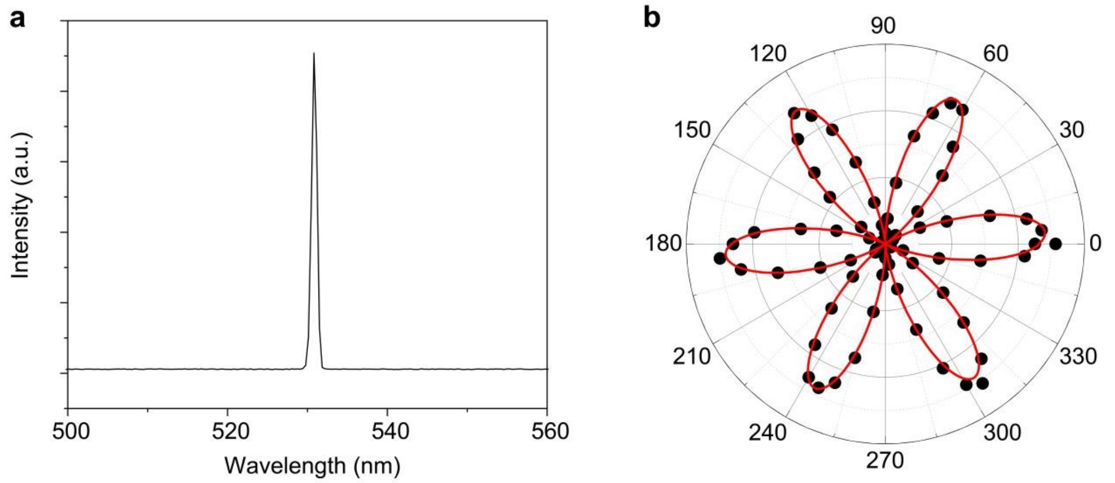

Fig. S1 │ SHG results of the 3R-MoS_2. a, SHG spectrum of the 3R-MoS_2. **b**, Polarization angle dependence of SHG intensity.

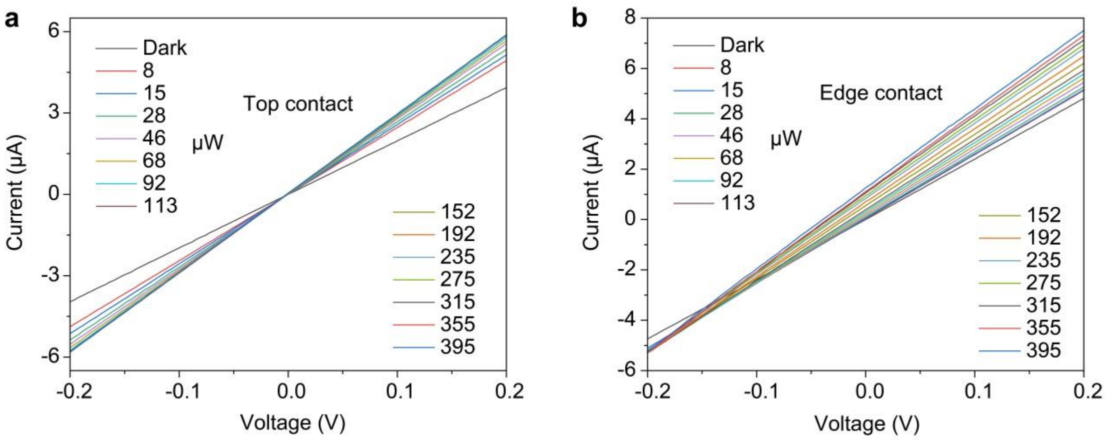

Fig. S2 │ Characteristics of the BPVE in both the TC and EC 3R-MoS_2 devices. a, b, *I*-*V* curves of the TC (**a**) and EC (**b**) device under illumination of different powers.

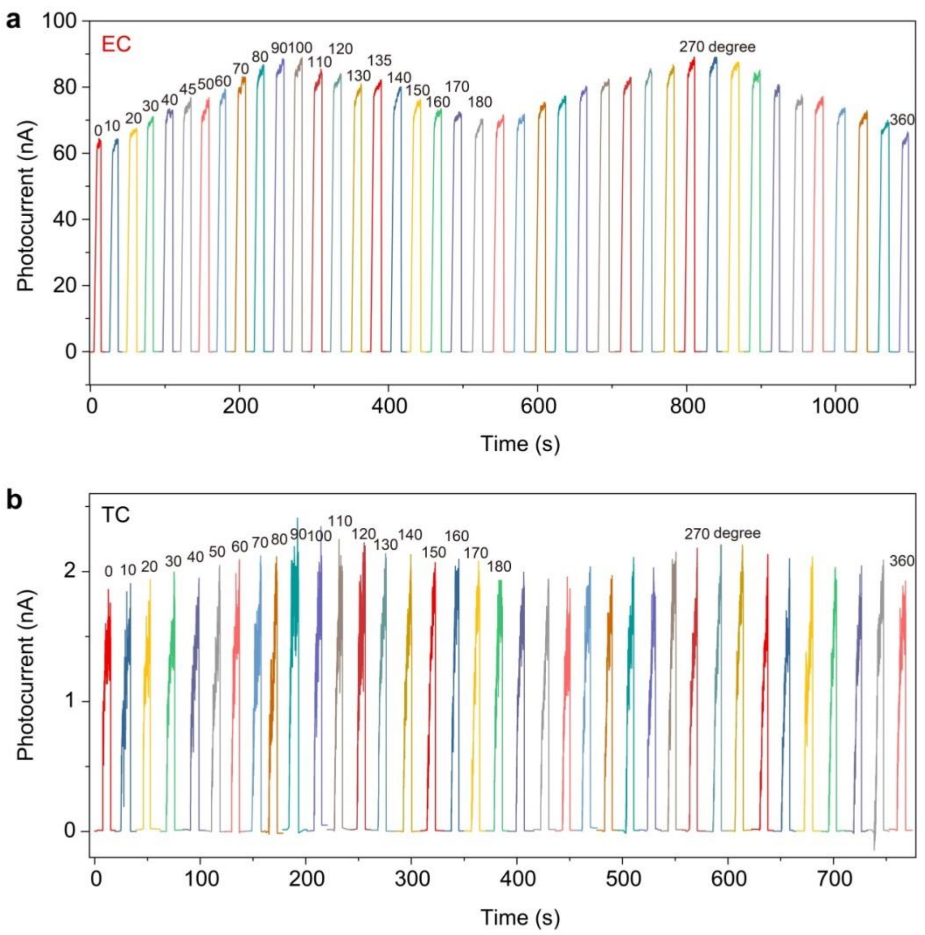

Fig. S3 │ Laser polarization-dependent photocurrent for both EC and TC 3R-MoS_2 devices. **a, b,** Time-dependent photocurrent curves of the EC (**a**) and TC (**b**) device under illumination of different laser polarizations. The laser wavelength is 532 nm and the power is 10 μW.

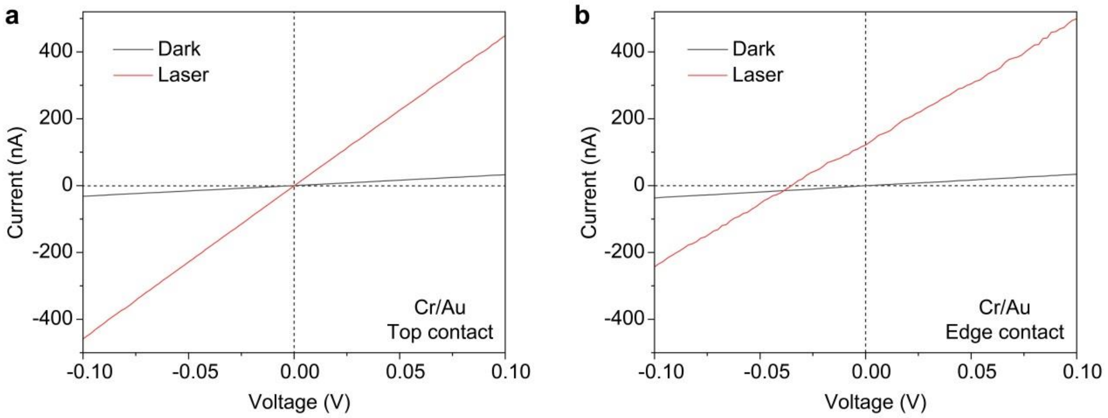

Fig. S4 │ Characteristics of the BPVE in Cr/Au-based 3R-MoS_2 devices. **a**, **b**, The *I*-*V* curves of the Cr/Au electrode-based TC 3R-MoS_2 device (**a**) and EC 3R-MoS_2 device (**b**). The laser wavelength is 488 nm and the power is 395 μW.

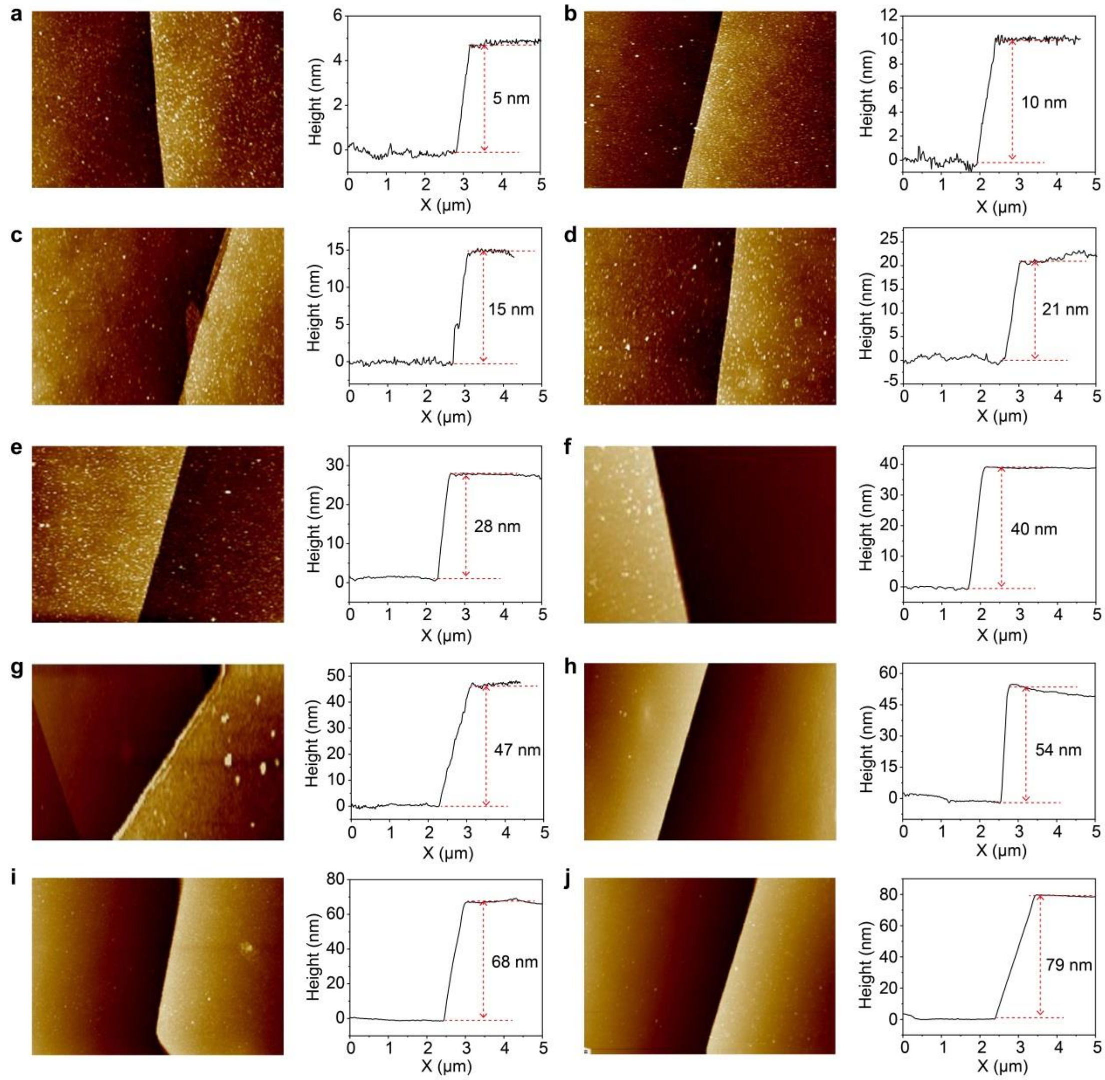

Fig. S5 │ Characteristics of the thickness in EC 3R-MoS_2 devices. a-i, AFM results of the EC 3R-MoS_2 devices with different thicknesses of (**a**) 5, (**b**) 10, (**c**) 15, (**d**) 21, (**e**) 28, (**f**) 40, (**g**) 47, (**h**) 54, (**i**) 68, and (**j**)79 nm.

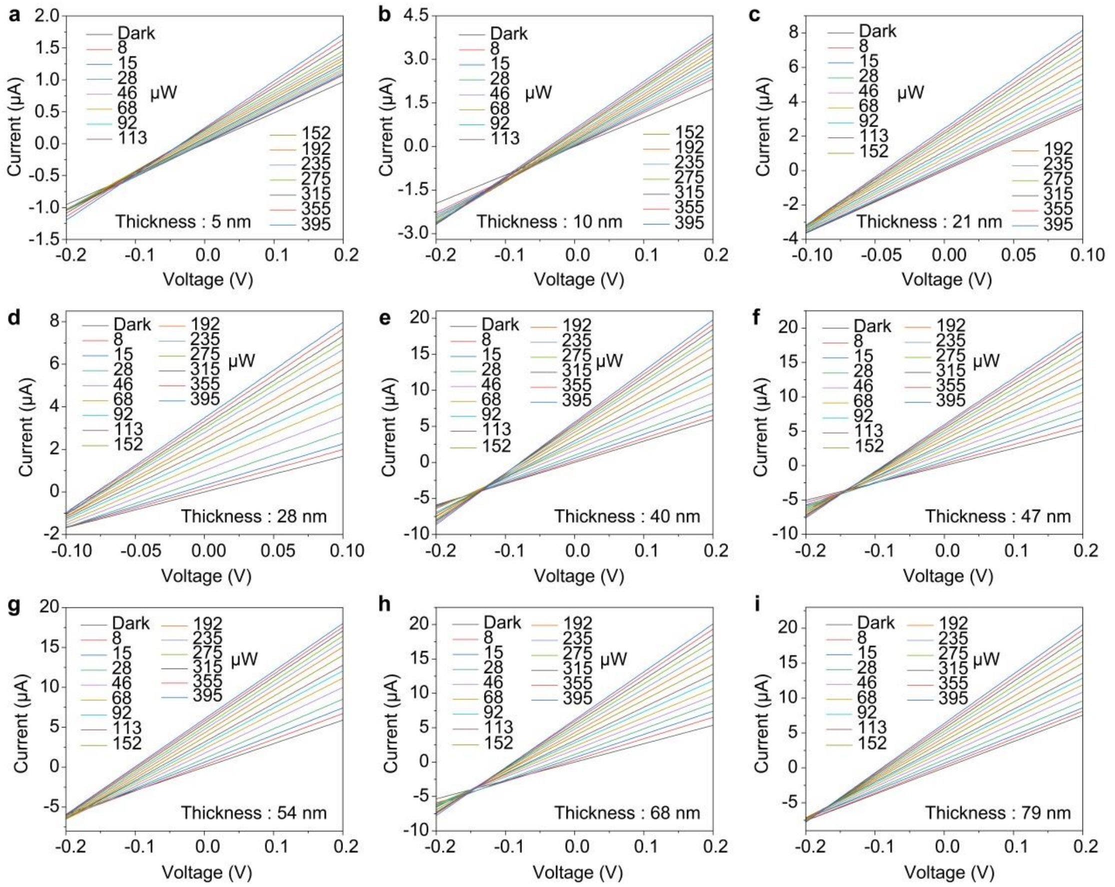

Fig. S6 │ Characteristics of the BPVE in EC 3R-MoS_2 devices. a-i, *I-V* curves of the EC 3R-MoS_2 devices with different thicknesses of (**a**) 5, (**b**) 10, (**c**) 21, (**d**) 28, (**e**) 40, (**f**) 47, (**g**) 54, (**h**) 68, and (**i**) 79 nm.

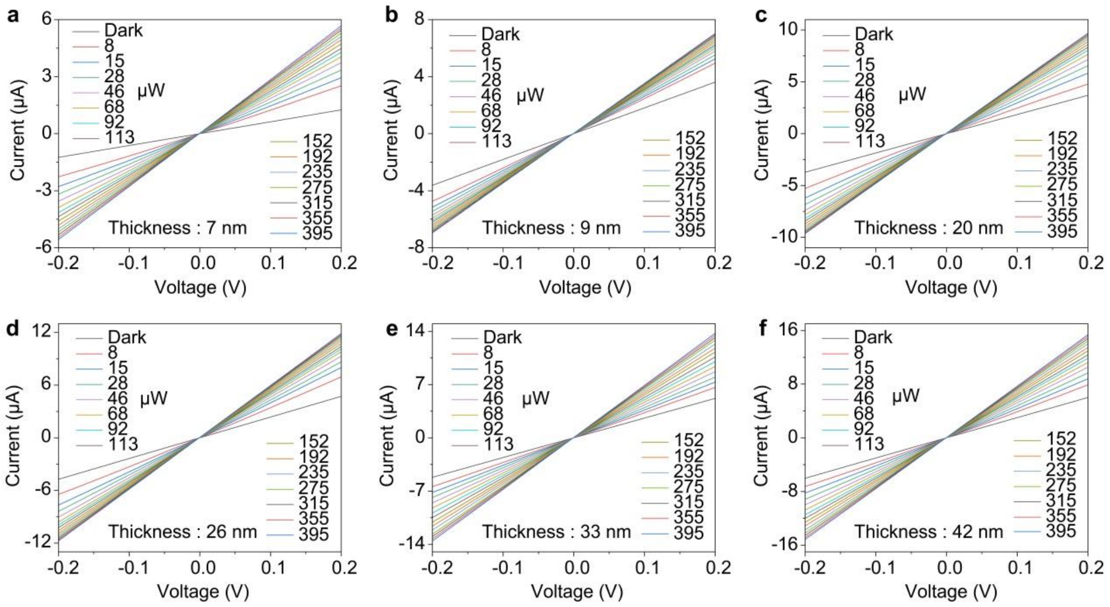

Fig. S7 │ Characteristics of the BPVE in TC 3R-MoS_2 devices. a-f, *I-V* curves of the EC 3R-MoS_2 devices with different thicknesses of (**a**) 7, (**b**) 9, (**c**) 20, (**d**) 26, (**e**) 33, and (**f**) 42 nm.

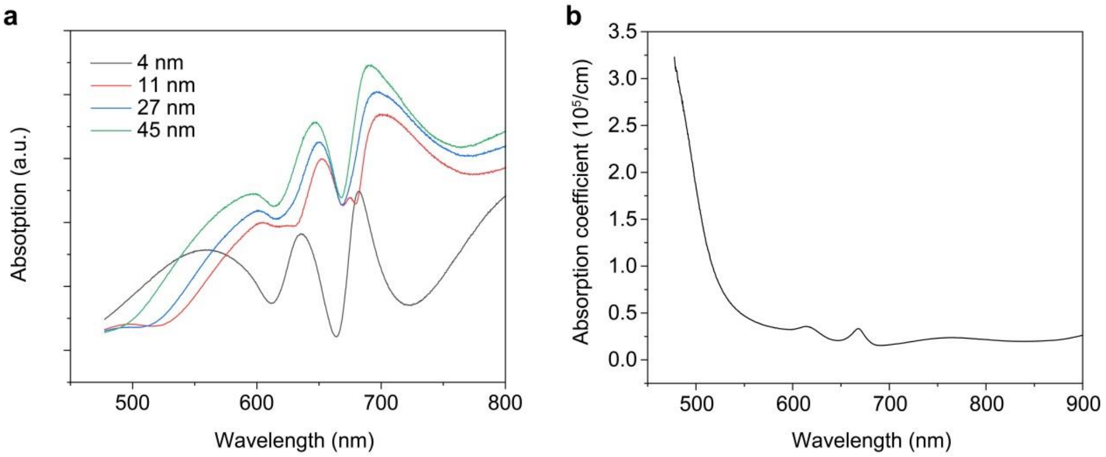

Fig. S8 │ Characteristics of the photoabsorption in 3R-MoS_2. Absorption results of the 3R-MoS_2 with different thicknesses (**a**). Calculated absorption coefficient of the 3R-MoS_2 with 45 nm-thick (**b**).

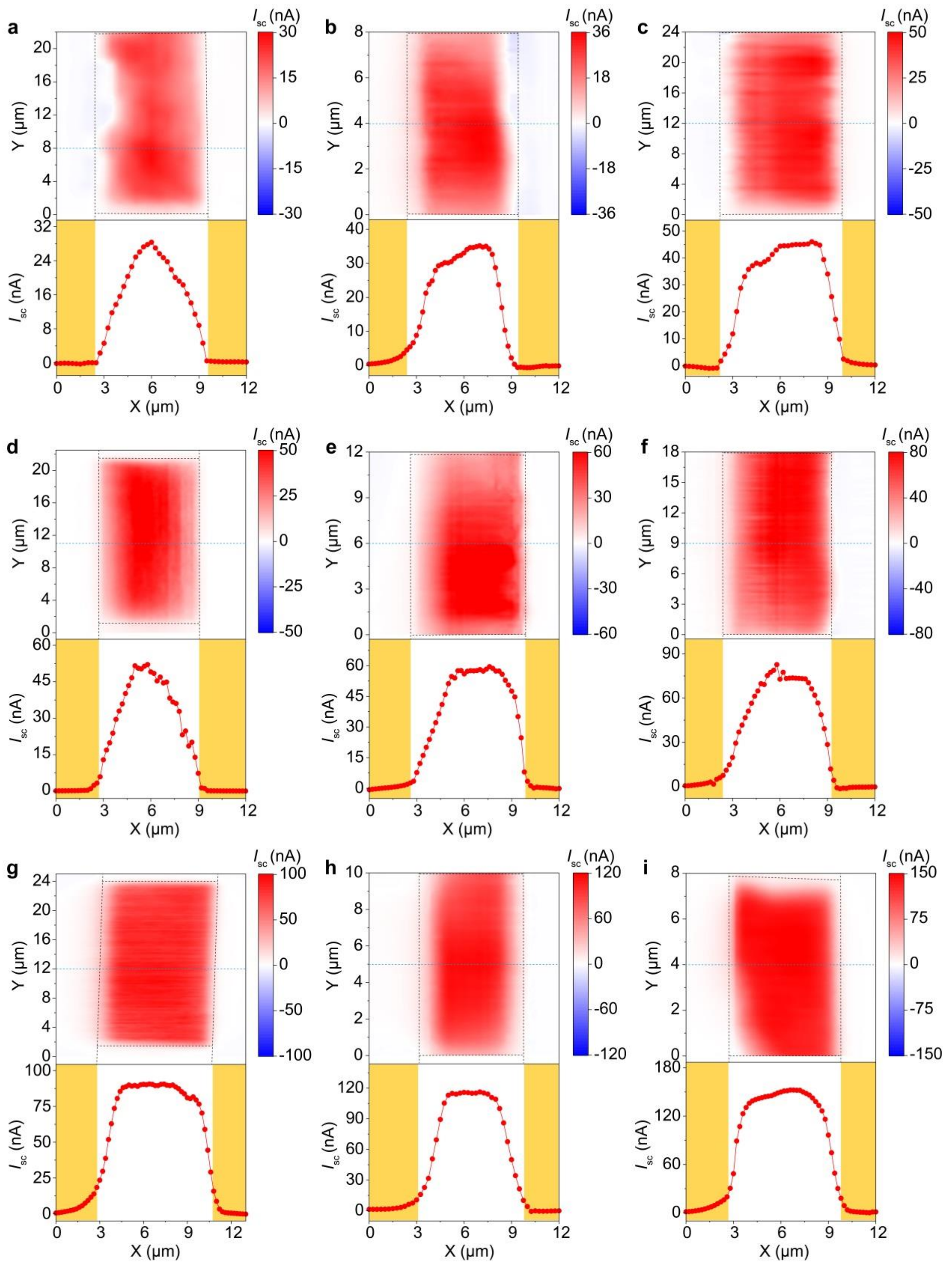

Fig. S9 │ Spatial photocurrent mappings of EC 3R-MoS_2 devices. a-i, The spatial photocurrent mappings and the illumination position dependences of I_{sc} corresponding to the marked dotted light-blue lines for the EC 3R-MoS_2 devices with thicknesses of (**a**) 10, (**b**) 14, (**c**) 18, (**d**) 20, (**e**) 24, (**f**) 31, (**g**) 38, (**h**) 49, and (**i**) 67 nm, respectively. The laser wavelength is 532 nm and the power is 3 μW.

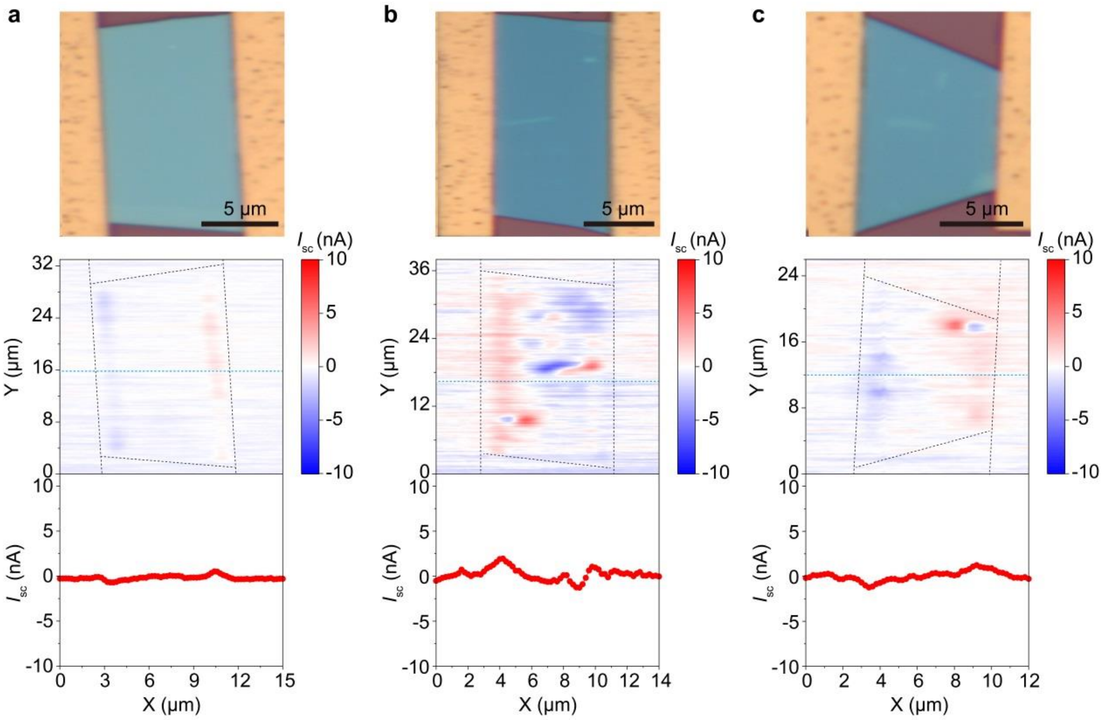

Fig. S10 │ Spatial photocurrent mappings of EC 2H-MoS_2 devices. a-c, Device optical micrograph, the corresponding photocurrent mapping and the illumination position dependence of I_{sc} along the marked dotted light-blue line for the EC 2H-MoS_2 devices with various shapes and thicknesses of (**a**) 10, (**b**) 13, (**c**) 12 nm, respectively. The laser wavelength is 532 nm and the power is 3 μW.

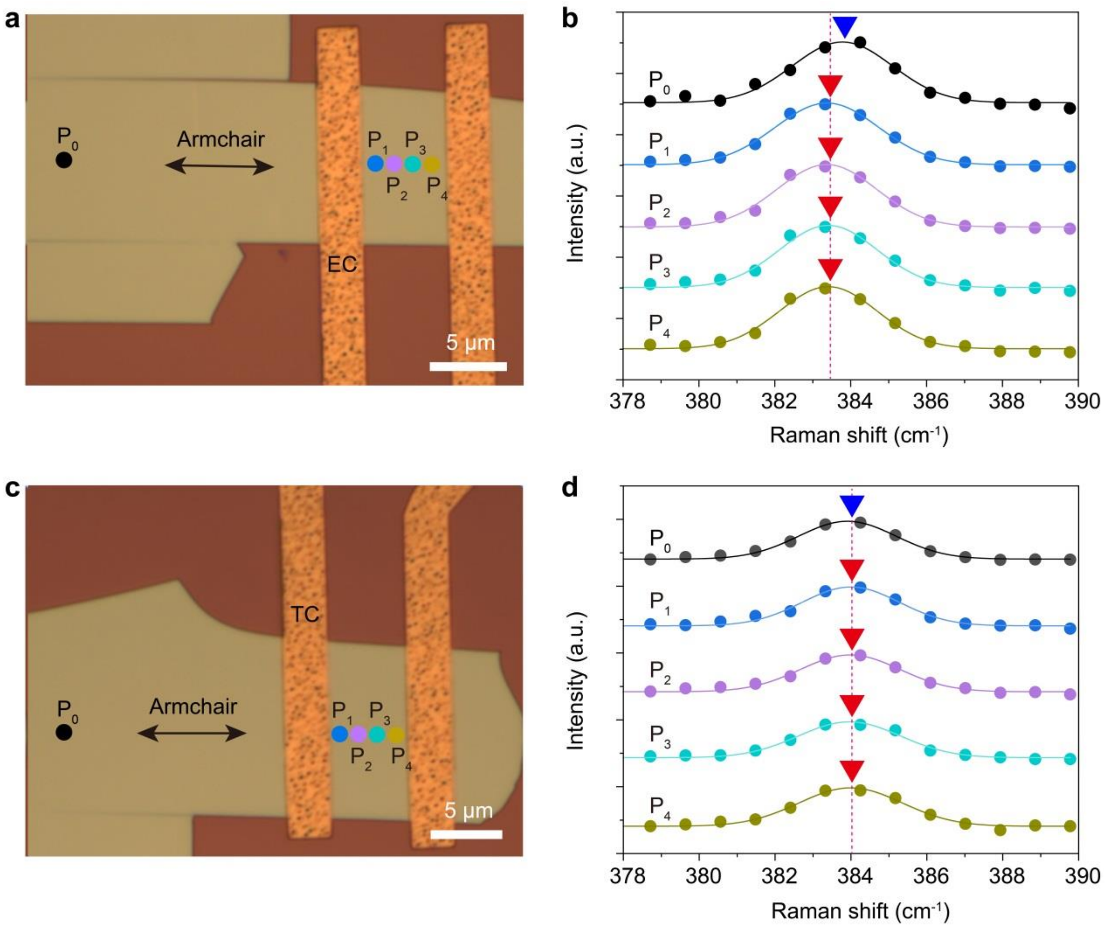

Fig. S11 │ Characteristics of the contact strain in the EC and TC devices. a, c, Optical micrographs of the EC (**a**) and TC (**c**) devices, P_0, P_1, P_2, P_3, and P_4 represent the measured positions. **b, d,** Raman peaks of E_{2g}^1 in different positions of the EC (**b**) and TC (**d**) devices.

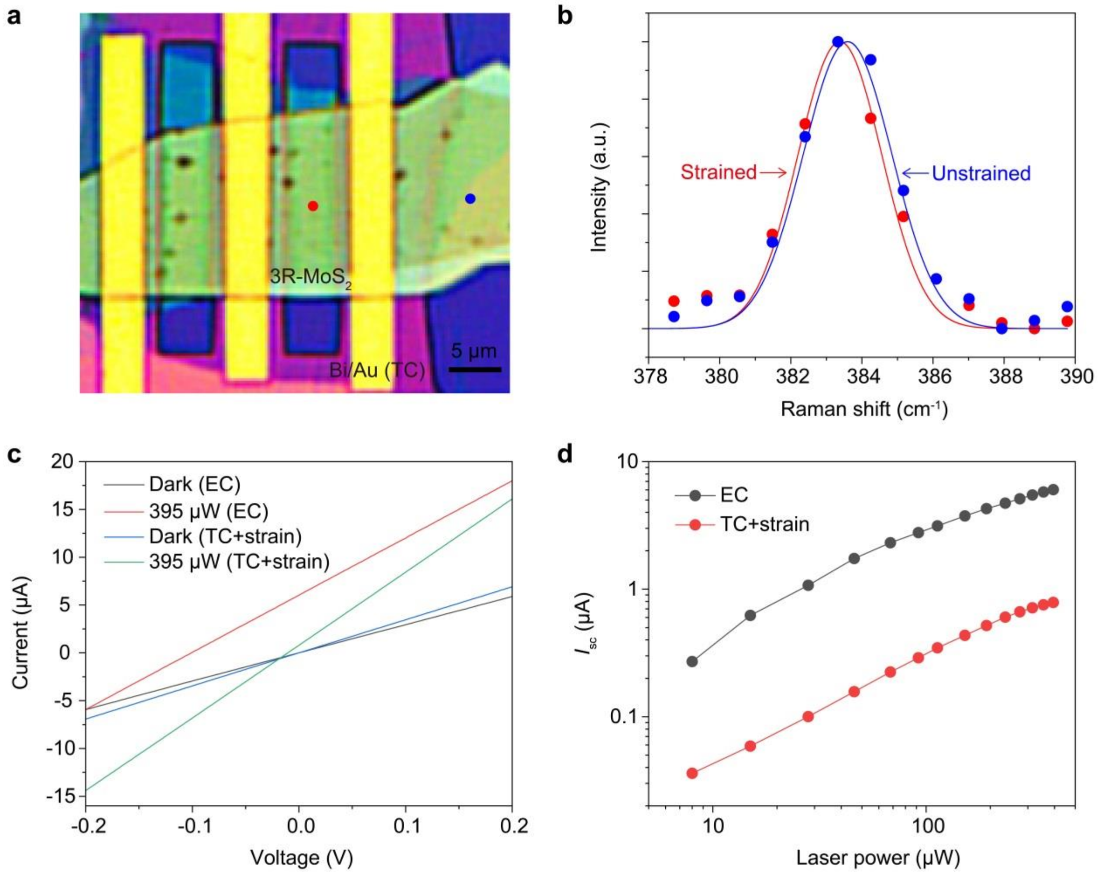

Fig. S12 │ Characteristics of the BPVE in external strained TC 3R-MoS_2 device. a, Optical micrograph of the strained TC 3R-MoS_2 device. **b**, Raman peak of E_{2g}^1 in unstrained (blue) and strained (red) region of the 3R-MoS_2 device (as marked in **a**). **c**, The *I*-*V* curves of the EC (54 nm-thick) and external strained TC (57 nm-thick) 3R-MoS_2 devices. **d**, Laser power-dependence of the BPVE photocurrent for EC and externally strained TC 3R-MoS_2 devices. The laser wavelength is 488 nm.

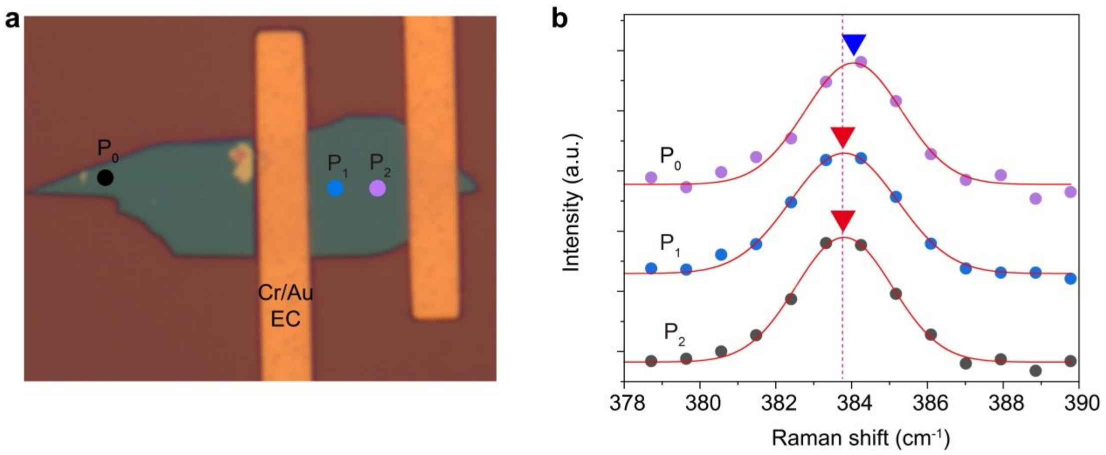

Fig. S13 │ Characteristics of the contact strain in the EC device of the Cr/Au electrodes. a, Optical micrograph of the device, P_0, P_1, and P_2 represent the measured positions. **b,** Raman peaks of E_{2g}^1 in different positions of the device, which correspond to ~ 0.13% of tensile strain at both P_1 and P_2 referencing to P_0.

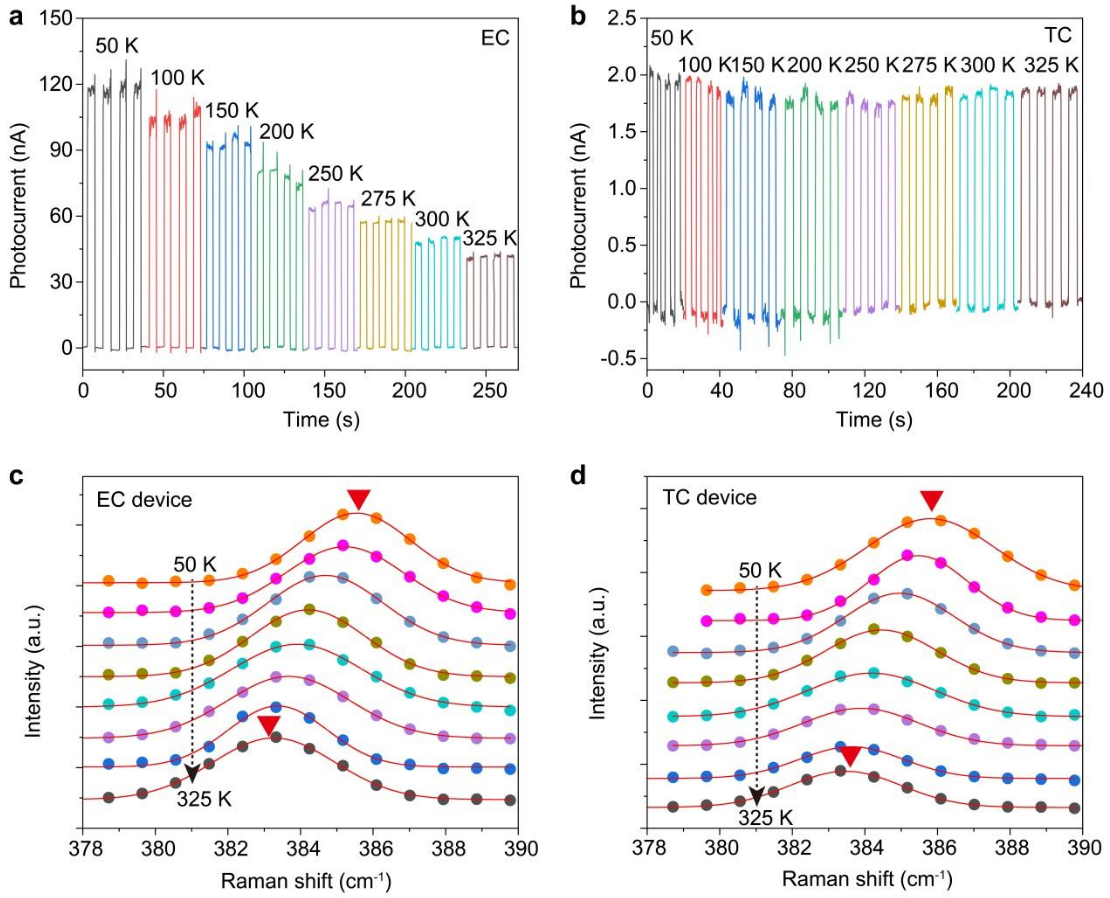

Fig. S14 │ Characteristics of the BPVE and Raman in the EC and TC devices. a, b, BPVE photocurrent in the EC (**a**) and TC (**b**) devices at different environmental temperatures. **c, d,** Raman spectroscopy in the EC (**c**) and TC (**d**) devices at different environmental temperatures.

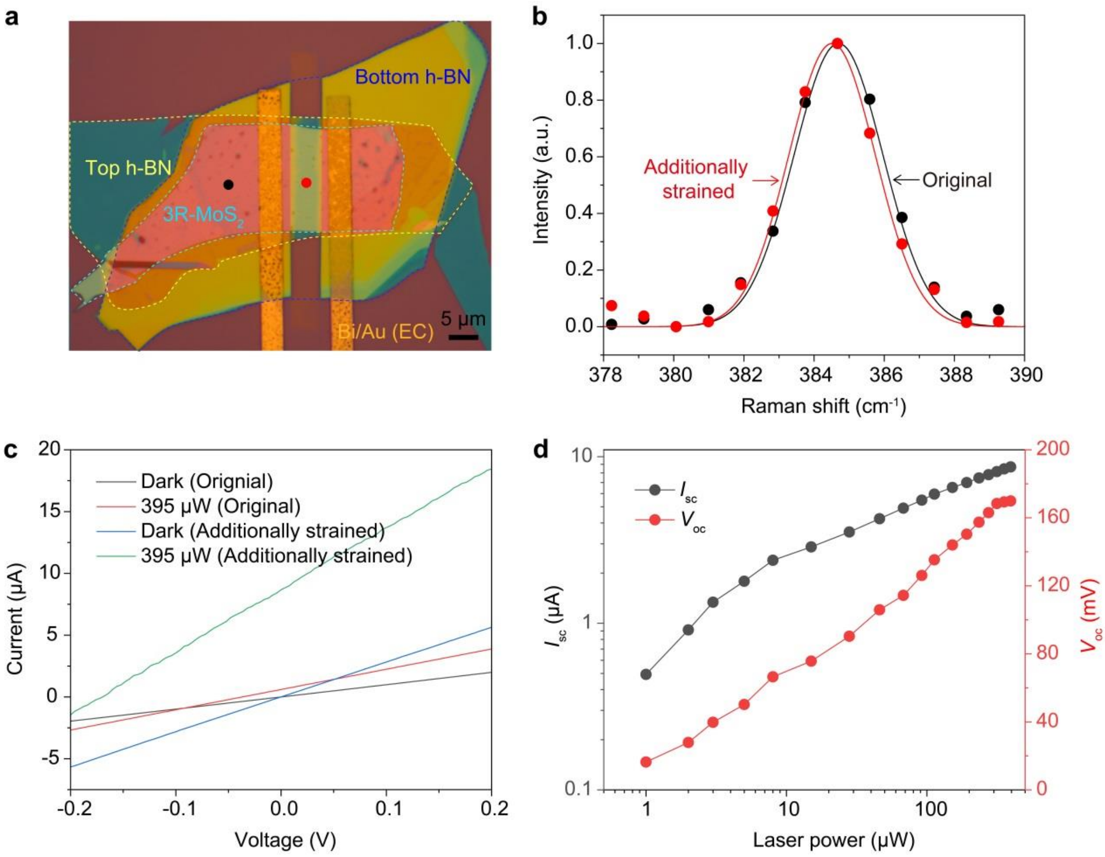

Fig. S15 │ Characteristics of the BPVE in strained EC 3R-MoS$_2$ device. **a**, Optical micrograph of the strained EC 3R-MoS$_2$ device. **b**, Raman peak of E_{2g}^{1} in original (black) and additionally strained (red) region of the 3R-MoS$_2$ device (as marked in **a**). **c**, The *I*-*V* curves of the original (10 nm-thick) and additionally strained EC 3R-MoS$_2$ devices. **d**, Laser power-dependence of the BPVE photocurrent and photovoltage for the additionally strained EC 3R-MoS$_2$ device. The laser wavelength is 488 nm.

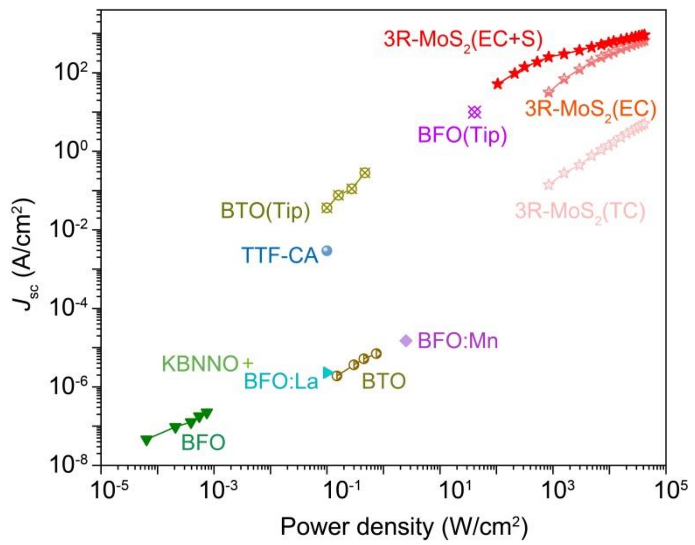

Fig. S16 │ BPVE J_{sc} comparison to non-TMD materials. Data for other materials (tip enhanced BFO (BFO(Tip))[1], tip enhanced BTO (BTO(Tip))[2], tetrathiafulvalene-p-chloranil (TTF-CA)[3], BFO:Mn[4], $[KNbO_3]_{1-x}[BaNi_{0.5}Nb_{0.5}O_{3-\delta}]_x$ (KBNNO)[5], La-substituted BFO (BFO:La)[6], BTO[7], BFO[8]) are shown as solid or open symbols and solid lines, respectively.

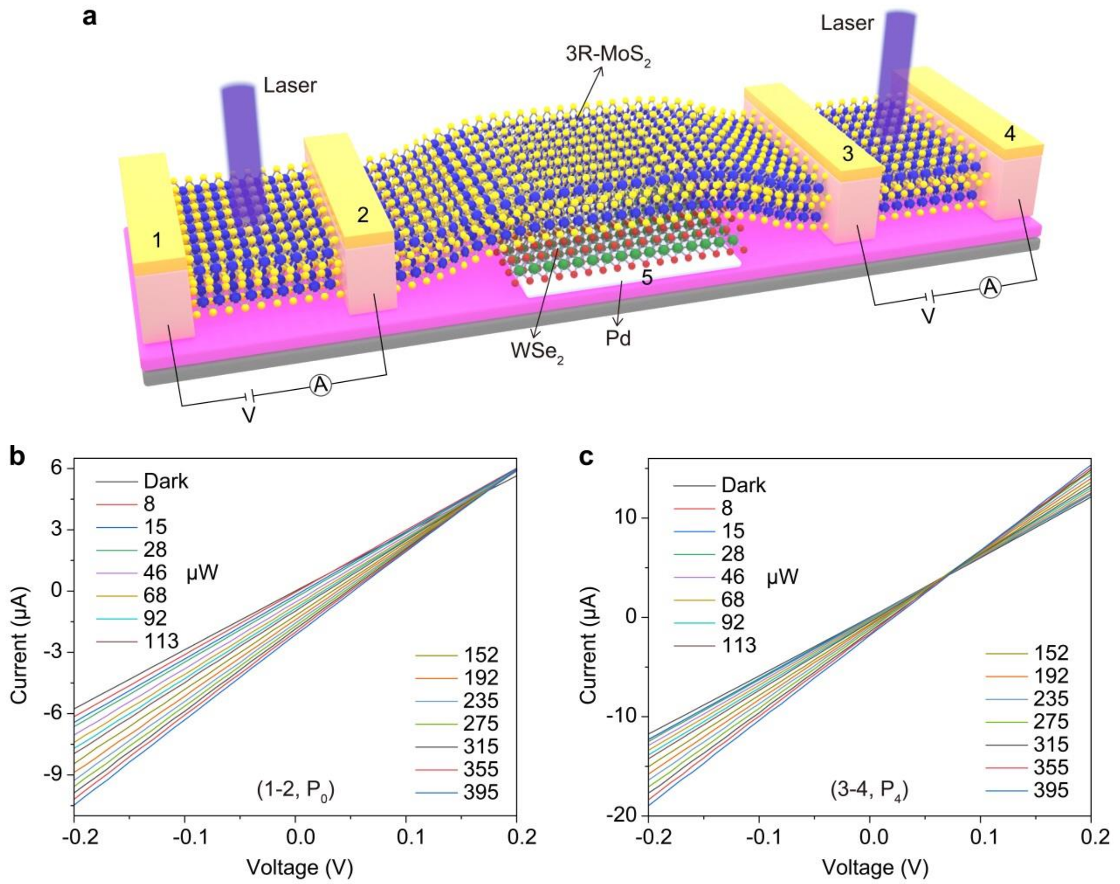

Fig. S17 │ Characteristics of the BPVE in EC 3R-MoS_2/WSe_2 heterojunction. a, Measurement schematic of the BPVE in the 3R-MoS_2 between electrodes 1-2 and 3-4. Note that the photocurrent contribution is simply from BPVE. **b**, **c**, The *I*-*V* curves of the electrodes 1-2 (**b**) and 3-4 (**c**) when illuminating at P_0 and P_4 positions as depicted in **a**, respectively. The laser wavelength is 488 nm.

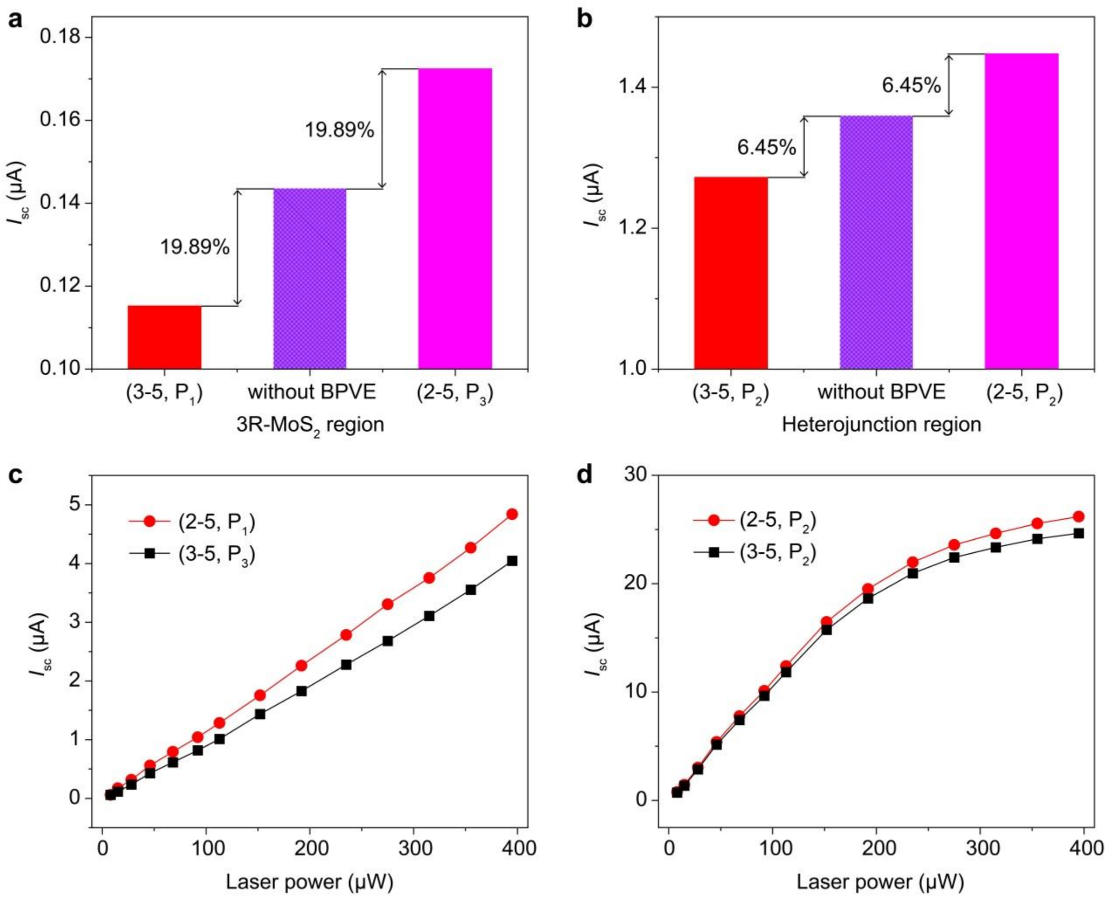

Fig. S18 │ BPVE effect on PVE performance in EC 3R-MoS_2/WSe_2 heterojunction. a, **b**, Positive and negative BPVE contributions on the PVE performance illuminated on the 3R-MoS_2 (**a**), and the heterojunction (**b**) at 15 μW. The BPVE contribution ratio is calculated based on the equation of $((I_{sc}(2\text{-}5)\text{-}I_{sc}(3\text{-}5))/((I_{sc}(2\text{-}5)+I_{sc}(3\text{-}5))$. **c**, **d**, The laser power dependence of the I_{sc} for electrodes 2-5 and 3-5 when illuminating on the 3R-MoS_2 (**c**), and the heterojunction (**d**).

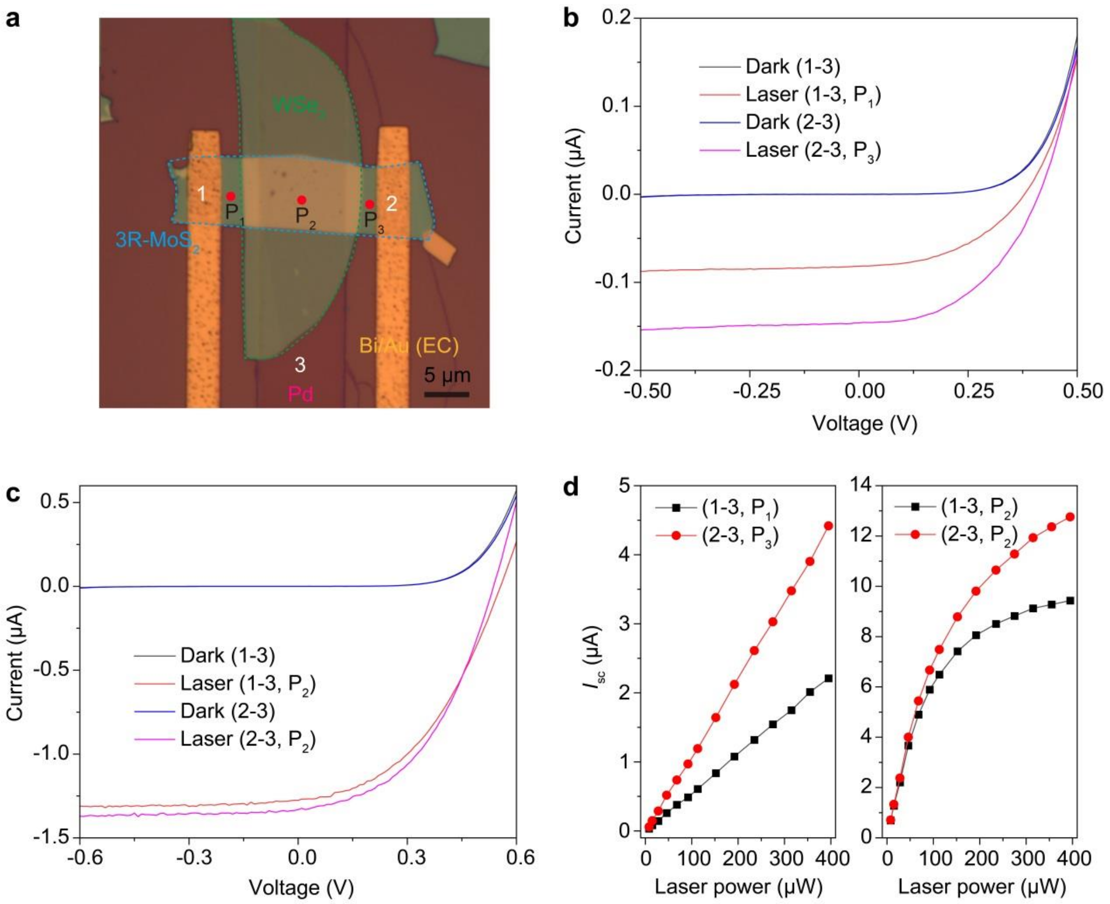

Fig. S19 │ PVE performance of EC 3R-MoS_2/WSe_2 heterojunction. **a**, Optical micrograph of the 3R-MoS_2/WSe_2 heterojunction device. **b**, **c**, The *I*-*V* curves of the electrodes 1-3 and 2-3 under dark and laser illumination, when the laser is illuminated on the 3R-MoS_2 (**b**), and the heterojunction (**c**). The laser wavelength is 488 nm, and the power is 15 μW. **d**, The laser power dependence of the I_{sc} for electrodes 1-3 and 2-3 when illuminating on the 3R-MoS_2 (left), and the heterojunction (right). The thickness of the 3R-MoS_2 and WSe_2 is 8 and 9 nm, respectively.

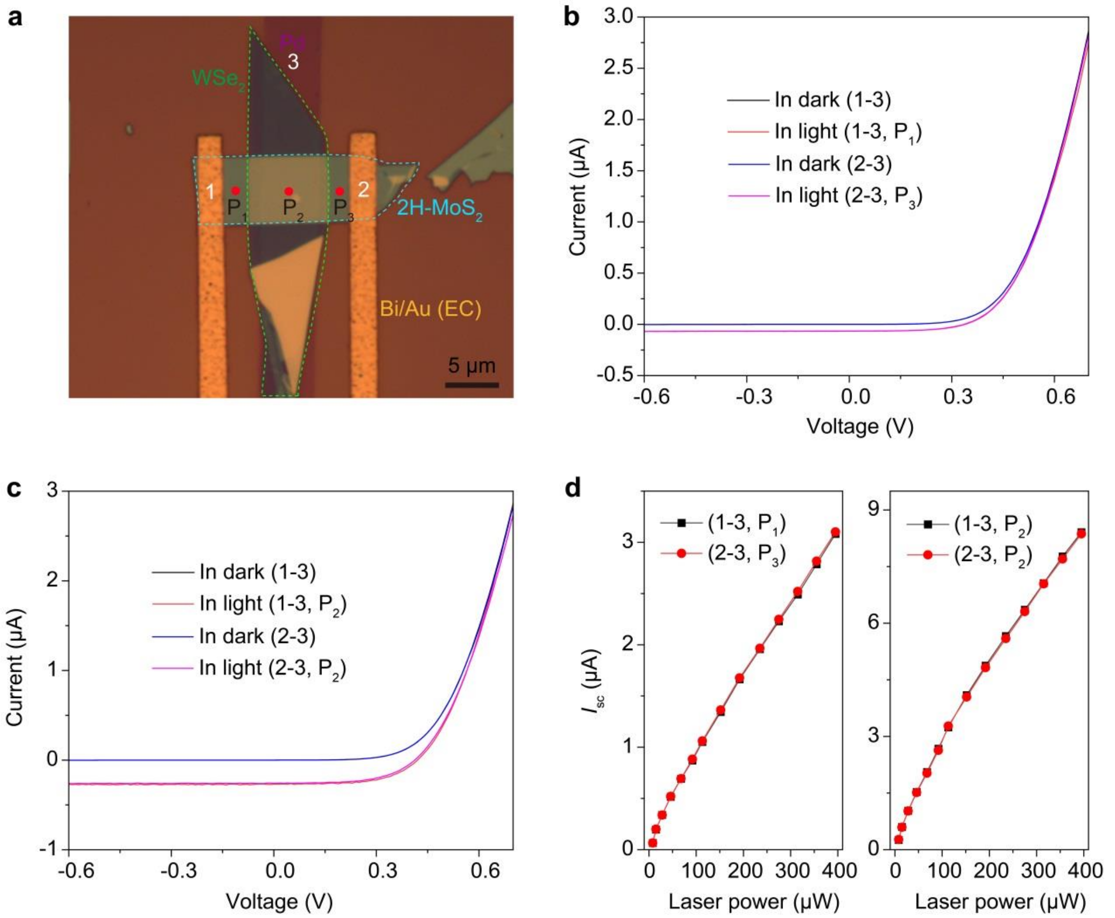

Fig. S20 │ PVE performance of EC 2H-MoS_2/WSe_2 heterojunction. **a**, Optical micrograph of the 2H-MoS_2/WSe_2 heterojunction device. **b**, **c**, The *I*-*V* curves of the electrodes 1-3 and 2-3 under dark and laser illumination, when the laser is illuminated on the 2H-MoS_2 (**b**), and the heterojunction (**c**). The laser wavelength is 488 nm, and the power is 15 μW. **d**, The laser power dependence of the I_{sc} for electrodes 1-3 and 2-3 when illuminating on the 2H-MoS_2 (left), and the heterojunction (right). The thickness of the 2H-MoS_2 and WSe_2 is 8 and 4 nm, respectively.

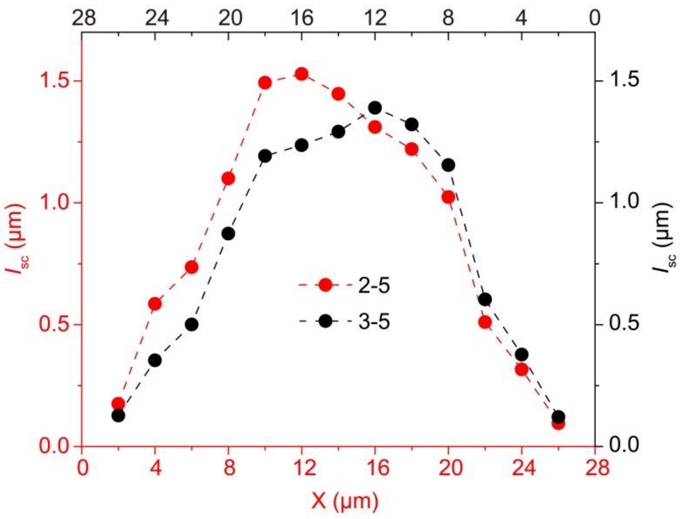

Fig. S21 │ Illumination position dependence of photocurrent in EC 3R-MoS_2/WSe_2 heterojunction. The laser wavelength is 488 nm, and the power is 15 μW.

References

1. Alexe, M. & Hesse, D. Tip-enhanced photovoltaic effects in bismuth ferrite. *Nature Communications* **2**, 256 (2011).

2. Spanier, J. E. et al. Power conversion efficiency exceeding the Shockley-Queisser limit in a ferroelectric insulator. *Nature Photonics* **10**, 611-616 (2016).

3. Nakamura, M. et al. Shift current photovoltaic effect in a ferroelectric charge-transfer complex. *Nature Communications* **8**, 281 (2017).

4. Nakamura, M. et al. Shift current photovoltaic effect in a ferroelectric charge-transfer complex. *Nature Communications* **8**, 281 (2017).

5. Grinberg, I. et al. Perovskite oxides for visible-light-absorbing ferroelectric and photovoltaic materials. *Nature* **503**, 509-512 (2013).

6. You, L. et al. Enhancing ferroelectric photovoltaic effect by polar order engineering. *Science Advances* **4**, eaat3438 (2018).

7. Zenkevich, A. et al. Giant bulk photovoltaic effect in thin ferroelectric $BaTiO_3$ films. *Physical Review B* **90**, 161409 (2014).

8. Ji, W., Yao, K. & Liang, Y. C. Bulk photovoltaic effect at visible wavelength in epitaxial ferroelectric $BiFeO_3$ thin films. *Advanced Materials* **22**, 1763-1766 (2010).
